# EP4 stimulation promotes cell adhesion and migration via IL-6 signaling in oral squamous cell carcinoma

**DOI:** 10.1016/j.jphyss.2026.100057

**Published:** 2026-01-15

**Authors:** Wakana Fukae, Soichiro Ishikawa, Yu Iida, Akane Nagasako, Michiko Endo, Chihiro Hayashi, Kagemichi Nagao, Sneri Oguri, Takayuki Fujita, Utako Yokoyama, Kenji Mitsudo, Yoshihiro Ishikawa, Masanari Umemura

**Affiliations:** a Cardiovascular Research Institute, Yokohama City University Graduate School of Medicine, 3-9 Fukuura, Kanazawa-ku, Yokohama-shi, Kanagawa 236-0004, Japan; b Department of Oral and Maxillofacial Surgery, Yokohama City University Graduate School of Medicine, 3-9 Fukuura, Kanazawa-ku, Yokohama-shi, Kanagawa 236-0004, Japan; c Department of Clinical Oral Oncology, National hospital organization, Hokkaido Cancer Center, 2-chome, Kikusui 4-jo, Shiroishi-ku, Sapporo-shi, Hokkaido 003-0804 3-54, Japan; d Department of Neurosurgery, Yokohama City University School of Medicine Graduate School of Medicine, 3-9 Fukuura, Kanazawa-ku, Yokohama-shi, Kanagawa 236-0004, Japan; e Department of Neurosurgery, Yokohama City Minato Red Cross Hospital, 3-12-1 Shinyamashita, Naka-ku, Yokohama-shi, Kanagawa 231-0801, Japan; f Department of Physiology, Fukuoka University, 7-45-1 Nanakuma, Jonan-ku, Fukuoka-shi 814-0180, Japan; g Department of Physiology, Tokyo Medical University, 1-5-45 Yushima, Bunkyo-ku, Tokyo, Japan; h Yokohama City University President, Japan

**Keywords:** Prostaglandin E₂ (PGE₂), EP4, Interleukin-6 (IL-6), Oral squamous cell carcinoma (OSCC), Cell adhesion, Cell migration

## Abstract

Oral squamous cell carcinoma cell (OSCC) comprises malignant neoplasms arising within the oral cavity. Early-stage detection is associated with favorable prognosis, whereas progression to advanced stages with lymph node metastasis significantly worsens outcomes. We previously reported that the prostaglandin E₂ (PGE₂) receptor EP4 regulates OSCC migration. RNA sequencing reanalysis suggested that EP4 stimulation is strongly associated with cell migration and adhesion, with interleukin-6 (IL-6) emerging as a central mediator of these processes. In OSCC cells, ONO-AE1–437 (EP4 agonist) increased IL-6 mRNA expression and protein secretion. EP4-overexpressing cells showed increased IL-6 expression without stimulation, further enhanced by ONO-AE1–437 or PGE₂. xCELLigence demonstrated that PGE₂ promoted cell adhesion, which was suppressed by ONO-AE3–208 (EP4 antagonist) and Tocilizumab (IL-6 inhibitor). Scratch and transwell assays revealed enhanced migration under PGE₂ and ONO-AE1–437, blocked by IL-6 inhibition. These results suggest that EP4 promotes cell adhesion and migration through IL-6 in OSCC cells. (147 words)

## Highlights

• EP4 stimulation increased the mRNA expression and protein secretion of IL-6.
• Overexpression of EP4 elevated IL-6 mRNA expression and protein secretion.
• PGE₂ promotes cell adhesion and migration through EP4 and IL-6.

## Introduction

As a non-steroidal anti-inflammatory drug (NSAID) with acetylsalicylic acid as its active ingredient, aspirin is widely used as an analgesic and antipyretic. Aspirin has long been suggested to have preventive effects against various cancers, including oral and colorectal cancer [1], [2]. Aspirin irreversibly inhibits cyclooxygenase-2 (COX-2), an inducible enzyme involved in inflammation and carcinogenesis, thereby reducing the levels of the lipid mediator prostaglandin E₂ (PGE₂) and contributing to cancer prevention [3]. COX-2 is overexpressed in a variety of cancers, and its upregulation has also been reported in oral squamous cell carcinoma (OSCC) [4]. PGE₂ production in OSCC is COX-2-dependent, and PGE₂ plays a key role in tumor progression, invasion, immune suppression, and angiogenesis [5], [6]. Therefore, PGE₂ receptors (EP receptors) may be a potential target for the prevention and treatment of OSCC.

PGE₂ is a major lipid mediator (autacoid) involved in physiological processes such as inflammation, pain, and fever, and exerts its effects by binding to G protein-coupled receptors on the cell membrane [7]. There are four subtypes of PGE₂ receptors: EP1, EP2, EP3, and EP4. EP1 is expressed in the nervous system and muscle tissue and is involved in pain and inflammation. EP2 regulates vasodilation in vascular smooth muscle and the kidney, modulates inflammatory responses, and provides neuroprotection. EP3 is associated with intestinal motility and fever responses, while EP4 has attracted attention for its roles in immune responses, inflammation, bone formation, pain control, and especially in cancer development and progression [8]. In fact, EP4 overexpression has been shown to promote the proliferation and invasion of colon cancer cells, contributing to tumor growth and metastasis [9]. Among the four PGE₂ receptors, EP3 and EP4 are reported to be highly expressed in OSCC [10].

OSCC, the predominant malignant tumor of the oral cavity, has poor prognosis in advanced cases, in which distant metastasis is a major determinant of patient outcome [11], [12]. Metastasis enables tumor growth in distant organs, leading to poor prognosis and reduced treatment efficacy [13]. Metastasis is initiated when cancer cells lose cell–cell adhesion molecules, weaken intercellular junctions, and detach from the primary tumor [14]. Enhanced migratory ability allows cancer cells to invade the surrounding stroma and migrate into blood and lymphatic vessels [15]. Therefore, regulation of cell adhesion and migration represents a key molecular basis for metastasis, and elucidating the mechanisms of adhesion and migration in cancer cells could contribute to the development of novel cancer therapies.

Our previous study identified a novel pathway in which EP4 stimulation induces the opening of the plasma membrane calcium channel Orai1, resulting in Ca²⁺ influx and promoting migration of OSCC cells [16]. Additionally, EP4 has been suggested to promote migration via calmodulin-like protein 6 (CALML6) in OSCC cells [17]. However, the detailed intracellular signaling pathways and molecular mechanisms underlying EP4-induced migration remain incompletely understood.

In the present study, reanalysis of RNA sequencing data from OSCC cells revealed that EP4 stimulation activated pathways related to the extracellular matrix (ECM) and associated cellular behaviors, including adhesion and migration. Furthermore, increased expression of interleukin-6 (IL-6) was identified as a potential key mediator of these functional changes. Interleukins are a group of cytokines mainly produced by immune cells and are involved in the regulation of immune responses, inflammation, and cell–cell interactions [18]. IL-6 is produced not only by immune cells (such as T cells, B cells, and macrophages) but also by fibroblasts and endothelial cells, and promotes immune cell activation and proliferation [19], [20]. In cancer, IL-6 is implicated in tumor progression, growth, metastasis, and treatment resistance, supporting the survival of cancer cells [21]. There have also been reports indicating that EP4 regulates IL-6 production [22]. However, the relationship between EP4 and IL-6 in OSCC cells remains unclear. In the present study, we demonstrated for the first time that EP4 regulates both cell migration and adhesion in OSCC cells through IL-6 signaling. While IL-6 is known to promote migration and induce adhesion molecules in OSCC cells, the involvement of EP4 in these processes had not been elucidated [23], [24].

Our findings provide direct evidence that EP4 modulates IL-6-mediated cell behavior in OSCC, highlighting a novel regulatory axis with potential therapeutic implications. Therefore, in this study, we focused on IL-6 to elucidate the effects of EP4 on cell adhesion and migration in OSCC cells and to investigate the intracellular signaling pathways involved in these processes.

## Material and methods

### Reagents

The EP4 agonist (ONO-AE1–437) and the EP4 antagonist (ONO-AE3–208) were kindly provided by Ono Pharmaceutical Co., Ltd. PGE₂ was purchased from Sigma-Aldrich, St. Louis, MO, USA [16], [17]. The COX-2 inhibitor (Celecoxib) was purchased from Tokyo Chemical Industry Co., Ltd. (Tokyo, Japan). The IL-6 inhibitor (Tocilizumab) was purchased from Chugai Pharmaceutical Co., Ltd. (Tokyo, Japan).

### Cell lines

The human oral tongue squamous cell carcinoma cell lines HSC-3, OSC-19, and SCC-25 were purchased from the Health Science Research Resources Bank, Japan Health Sciences Foundation [16], [17]. HSC-3 and OSC-19 are highly metastatic tumor cell lines, while SCC-25 is characterized by high EP4 expression. The human gingival fibroblast cell line (HGnF) was purchased from ScienCell Research Laboratories. HSC-3 and OSC-19 cells were cultured in Dulbecco’s Modified Eagle Medium (DMEM; FUJIFILM Wako Pure Chemical Corporation, Osaka, Japan) supplemented with 10 % fetal bovine serum (FBS; Nichirei Corporation, Tokyo, Japan) and 1 % penicillin-streptomycin. SCC-25 cells were cultured in D-MEM/Ham’s F-12 medium supplemented with 10 % FBS. HGnF cells were cultured in Fibroblast Medium (FM; ScienCell Research Laboratories, CA, USA).

### RNA-seq

RNA-seq data used in this study were originally obtained in our previous study [17]. In the present study, we reanalyzed these data focusing on IL-6-related pathways in oral squamous cell carcinoma under EP4 stimulation. Differentially expressed genes identified from RNA-seq (e.g., *p* < 0.05; |log₂FC| >0.58) were submitted to Metascape using default settings to perform pathway/GO enrichment, an enrichment term network, and PPI enrichment with Molecular Complex Detection (MCODE) [25]. The resulting PPI network was visualized using Cytoscape and the STRING app [26], [27]. For display, we showed only the largest connected component (LCC), omitting small disconnected components (<5 nodes). Centrality metrics (degree, betweenness centrality) were computed on the LCC using Tools → Analyze Network (default). Module extraction was performed using the MCODE algorithm implemented in Metascape to identify functional modules and hub proteins [28]. Furthermore, Gene Ontology (GO) enrichment analysis was conducted within the STRING app, and the biological processes (GO terms) associated with each MCODE cluster were annotated. The analysis was performed based on the "GO_MCODE.csv" file output from Metascape, with a particular focus on GO terms related to "cell adhesion" and "cell migration."

### Reverse transcription quantitative polymerase chain reaction (RT-qPCR)

Total mRNA was extracted from cells using ISOSPIN Cell & Tissue RNA kit (Nippon Gene, Tokyo, Japan) [16], [17], [29], [30]. RNA concentration was measured and adjusted using a NanoDrop 2000c (Thermo Scientific, Tokyo, Japan). Complementary DNA (cDNA) was synthesized from the extracted mRNA using a TaKaRa PCR Thermal Cycler Dice (TaKaRa Bio, Shiga, Japan). Quantitative PCR was performed using the StepOnePlus Real-Time PCR System (Applied Biosystems) with the following conditions: initial denaturation at 95 °C for 30 s, followed by 40 cycles consisting of denaturation at 95 °C for 5 s, annealing and extension at 60 °C for 30 s. Fluorescence signals were measured using TB Green to monitor amplification. Relative gene expression levels were determined using the 2^-ΔΔCT method, with 18S rRNA as the normalization control.

The primer sequences used in this study are listed below:

18S:

F: 5’-CTTTGGGCGGAAGACAGGTC-3’

R: 5’-CCATCCAATCGGTAGTAGCG-3’

IL-6:

F: 5’-CCAGGAGCCCAGCTATGAA-3’

R: 5’-TTCTGCCAGTGCCTCTTTG-3’

EP4:

F: 5’-CCGGCGGTGATGTTCATCTT-3’

R: 5’-CCCACATACCAGCGTGTAGAA-3’

### Enzyme-linked immunosorbent assay (ELISA)

The concentration of IL-6 in the culture supernatants was measured using a Human IL-6 Quantikine ELISA Kit (R&D Systems, Minneapolis, MN, USA) according to the manufacturer’s protocol [31]. HSC-3 and OSC-19 cells, as well as HSC-3 cells overexpressing EP4, were seeded at a density of 1 × 10⁵ cells per well in 6-well plates and cultured in serum-free medium for 24 h. The cells were then stimulated with PGE₂ (2 μM), ONO-AE1–437 (1 μM), or an ONO-AE3–208 (1 μM). After 1 h of stimulation, the culture supernatants were collected and centrifuged at 13,000 rpm for 10 min at 4 °C to remove cellular debris. The supernatants were stored at −80 °C until further analysis. The IL-6 concentration in each sample was determined by ELISA following the standard curve provided in the kit.

### Lentiviral transduction in vitro

Lentiviral constructs (scramble control and hPTGER4) were synthesized by Vector Builder (Chicago, IL, USA) and transduced into HSC-3 cells [17]. After 24 h, the culture medium was replaced, and stably transduced cells were selected using 0.15 µg/mL blasticidin. The transduction efficiency of the lentivirus was evaluated by RT-qPCR and western blotting.

### Western blotting

Western blot analyses were performed as previously described [32]. Briefly, whole-cell lysates were prepared using RIPA buffer (Thermo Scientific, IL, USA). Protein concentration was determined using the BCA Protein Assay (Thermo Scientific, IL, USA). Proteins were separated on 8 % SDS-PAGE and transferred to polyvinylidene fluoride membranes (Millipore, IPVH00010). To reduce non-specific bands, membranes were blocked with 0.3 % bovine serum albumin and 0.5 % skim milk for 30 min. The following primary antibodies were used; EP4 (Cayman Chemical, Ann Arbor, MI, USA); and GAPDH (Santa Cruz Biotechnology, CA, USA). After incubation with the appropriate HRP-conjugated secondary antibodies (anti-rabbit IgG or anti-mouse IgG, Cell Signaling Technology, MA, USA; 1:2000 dilution) for 1 h, chemiluminescence was detected using an ECL substrate (Bio-Rad Laboratories, Inc., CA, USA). The band intensities were quantified using ImageJ software (NIH Image, Bethesda, MD, USA).

### Cell viability assay

HSC-3 cells were seeded in 96-well plates at a density of 5.0 × 10³ cells per well. The cells were cultured in DMEM for 24 h. After the addition of Tocilizumab, the cells were incubated for an additional 24 h. Cell viability was measured using the Cell Counting Kit-8 (CCK-8) assay (Dojindo, Kumamoto, Japan) according to the manufacturer’s protocol [32].

### Cell adhesion assay

Cell adhesion was evaluated using the xCELLigence Real-Time Cell Analysis (RTCA) system (ACEA Biosciences, San Diego, CA, USA) [33]. Each well of an E-Plate 16 was coated with fibronectin (10 μg/mL in PBS) for 1 h at 37 °C. After removing the unbound fibronectin, the wells were washed once with PBS.

Cells were harvested and resuspended in serum-free medium. A total of 5.0 × 10⁴ cells were seeded per well. Immediately after seeding, cells were treated with PGE₂ (2 μM), ONO-AE3–208 (1 μM), or Tocilizumab (1 μg/mL). Electric impedance (Cell Index) was measured every 2 min for the first 30 min, followed by every 5 min up to 6 h. All Cell Index values were normalized to the value at the time of cell seeding (time zero). The degree of cell adhesion was quantified by comparing the Cell Index at specific time points (3 h after the start of measurement). Each condition was assayed in triplicate, and the experiments were repeated independently at least three times.

### Scratch assay

Phase-contrast images were acquired and analyzed using an ECLIPSE Ti microscope (Nikon Corporation, Tokyo, Japan) [17]. Cells were seeded in 24-well plates at a density of 2.0–3.0 × 10^5^ cells per well and cultured at 37 °C until reaching confluence. A sterile 1000 μL pipette tip was used to create a scratch in the cell monolayer, followed by washing with medium to remove detached cells.　Images were captured at 0 and 10 h after scratching, and cell migration was analyzed by measuring the migration distance.

### Transwell assay

Cells at a density of 2.0 × 10⁵ cells/mL were seeded into the upper chamber of Matrigel-coated polycarbonate Transwell inserts (QCM 24-Well Cell Invasion Assay, Cell Biolabs, Inc.) specifically designed for invasion assays [34]. Cells were suspended in serum-free medium in the upper chamber, while the lower chamber was filled with medium containing 2 % FBS as a chemoattractant. Cells were incubated at 37 °C for 24 h.　Non-invading cells on the upper surface of the membrane were removed using a cotton swab. Cells that had invaded to the lower surface of the membrane were fixed with 4 % paraformaldehyde and stained with crystal violet. The number of invaded cells was counted under a BZ-X800 all-in-one fluorescence microscope (Keyence Corporation, Osaka, Japan) in four randomly selected non-overlapping fields.

### Statistical analysis

Statistical analysis was performed using GraphPad Prism 9 software (GraphPad Software Inc., San Diego, CA, USA). After confirming that the data followed a normal distribution, two-tailed Student’s *t*-test was applied to determine the significance of differences between two groups of independent samples. Pearson’s correlation analysis was performed to determine the correlation between two variables. Comparisons among more than two groups were performed using one-way analysis of variance (ANOVA) followed by Tukey’s post hoc test. All *p*-values are indicated in the figure (*p < 0.05; **p < 0.01; ***p < 0.001; n.s., not significant).

## Results

### IL-6 emerges as a central hub gene downstream of EP4 signaling, associated with cell migration and adhesion

**Fig. 1:**
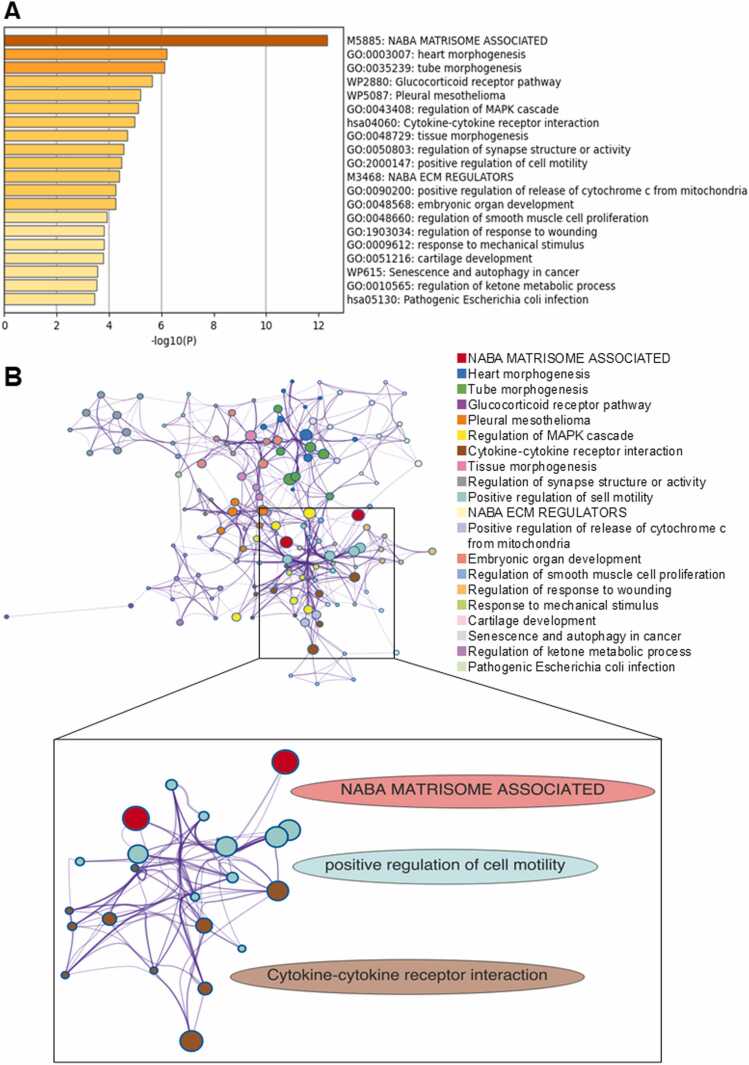

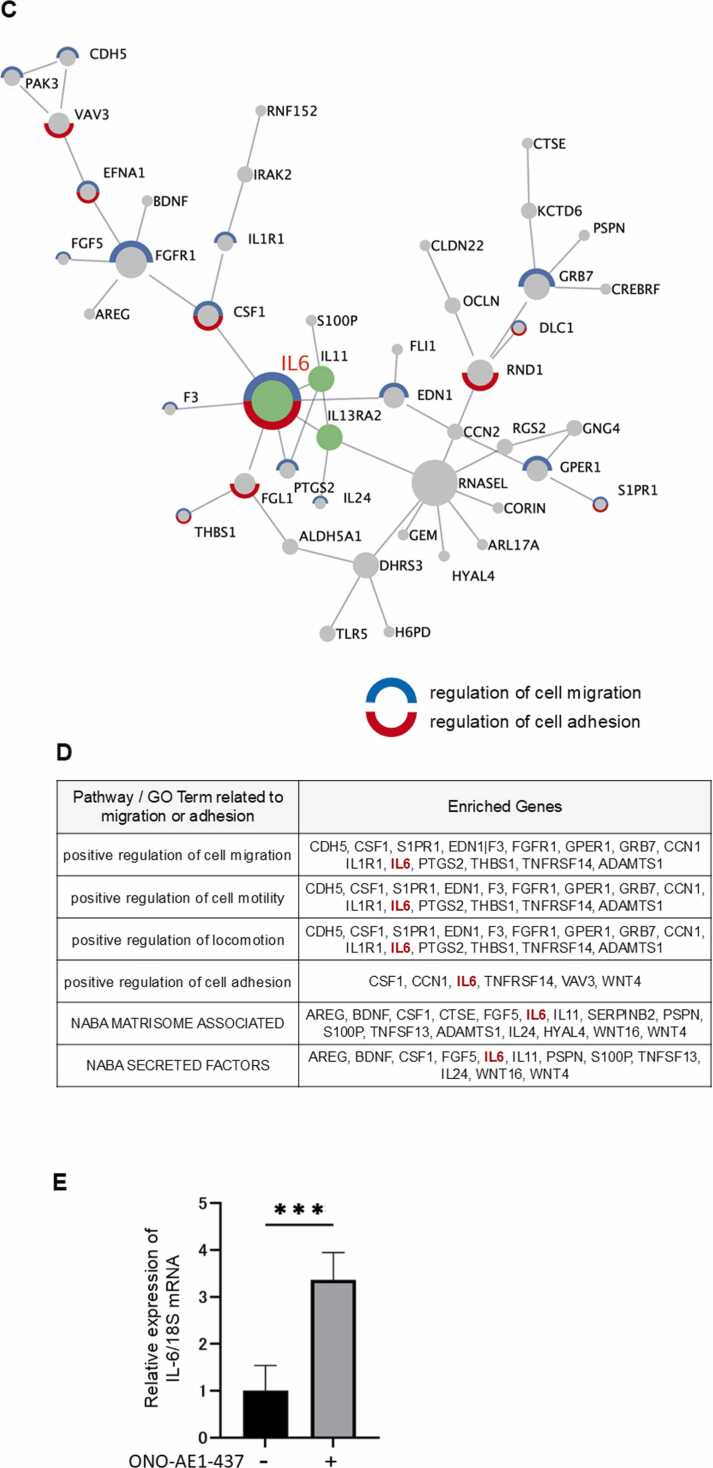
**RNA-seq analysis demonstrates increased IL-6 expression following EP4 stimulation.** RNA sequencing was performed to comprehensively analyze gene expression changes in HSC-3 cells treated with ONO-AE1–437 (1 µM) for 3 h. (A) Bar plot of enrichment (Metascape, default) generated from genes meeting the RNA-seq criteria (*p* < 0.05 and |log₂FC| ≥ 0.58). Top enriched terms from the differential gene expression list show extracellular matrix (NABA Matrisome associated) at the highest rank, together with cytokine-related and cell motility terms (values plotted as −log10(P)). (B) Enrichment network of EP4-stimulated RNA-seq. Upper panel: Overall enrichment network. Nodes represent enriched terms; edges connect term pairs with similarity > 0.3. Node colors indicate cluster IDs and size reflects hit count. Terms were selected by Metascape’s default sampling. Lower panel：Focused subnetwork of selected clusters：Focused subnetwork of selected clusters. From upper panel, only the terms belonging to the three clusters—NABA Matrisome associated, positive regulation of cell motility, and Cytokine–cytokine receptor interaction—were extracted and redrawn under the same thresholds and styling. Elliptical bands indicate cluster names. (C) A protein-protein interaction (PPI) network was constructed using Metascape. Node size reflects degree (number of edges). This revealed IL-6 as a hub in the PPI network, showing high degree (k = 7) and betweenness (0.286). Enrichment analysis of the network components identified genes involved in the regulation of cell adhesion and migration, which were visualized. The genes marked in blue are associated with the regulation of cell migration, while the genes marked in red are related to the regulation of cell adhesion. MCODE clustering of the PPI network identified three core clusters, one of which included IL-6 as a hub. Colored nodes indicate MCODE-extracted components. (D) Gene Ontology (GO) terms associated with this cluster included several terms related to cell migration and cell adhesion. (E) RNA-sequencing analysis of gene expression changes in HSC-3 cells treated with the EP4 agonist ONO-AE1–437 (1 μM) for 3 h (n = 4). Differentially expressed genes were identified based on normalized read counts obtained from comprehensive transcriptomic profiling. The IL-6 expression data shown were extracted from the RNA-sequencing results and are presented as a representative EP4-responsive gene.

Comprehensive reanalysis of our previously published RNA sequencing data from EP4-stimulated HSC-3 cells identified a set of significantly altered genes [17]. Enrichment analysis using Metascape for genes significantly upregulated by EP4 stimulation revealed that *NABA_MATRISOME_ASSOCIATED* was the top-ranked enriched term (Fig. 1**A**). These gene sets comprise structural and regulatory components of the ECM, suggesting that EP4 stimulation may influence the ECM microenvironment and related cellular behaviors, such as adhesion and migration [35]. Furthermore, enrichment of GO terms such as “positive regulation of cell motility” and “cytokine–cytokine receptor interaction” was observed. Moreover, the enrichment network showed dense inter-connections among ECM, motility, and cytokine clusters (Fig. 1**B, upper panel**); a focused subnetwork emphasized these links (Fig. 1**B, lower panel**). These results indicated potential involvement of cytokine-mediated regulation of the ECM and control of cell migration. PPI networks were generated for significantly altered genes using Metascape, revealing IL-6 as a hub showing high degree (k = 7) and high betweenness centrality (0.286). Furthermore, IL-6 was identified as a core component within functional modules extracted by MCODE analysis (Excel file named “GO_MCODE”). Enrichment analysis of the entire PPI network revealed that, within the MCODE-extracted module, IL-6 was the only gene co-annotated to both adhesion- and migration-related terms. CSF1, although annotated to both, was not captured by any MCODE component. (Fig. 1**C**). IL-6 is produced not only by immune cells but also by stromal and tumor cells, and has been implicated in cancer progression, metastasis, and treatment resistance [20], [22]. Reports also suggest that EP4 regulates IL-6 production [22]. According to The Human Protein Atlas, head and neck cancer cell lines (HSC-3 and SCC-25) express both EP4 and IL-6 at the RNA level (Fig. S1**, 2**).

These results support the notion that IL-6 serves as a key mediator of cell migration and adhesion. Consistent with these computational predictions, clustering analysis using MCODE also included IL-6 in several terms related to cell migration and adhesion, as well as ECM-related cellular dynamics (Fig. 1**D**). To experimentally validate these observations, IL-6 mRNA expression extracted from the RNA-sequencing dataset was increased in HSC-3 cells following treatment with the selective EP4 agonist ONO-AE1–437 (1 μM for 3 h) compared with control cells (Fig. 1**E**).

### Increase in IL-6 expression and secretion induced by EP4 stimulation

**Fig. 2:**
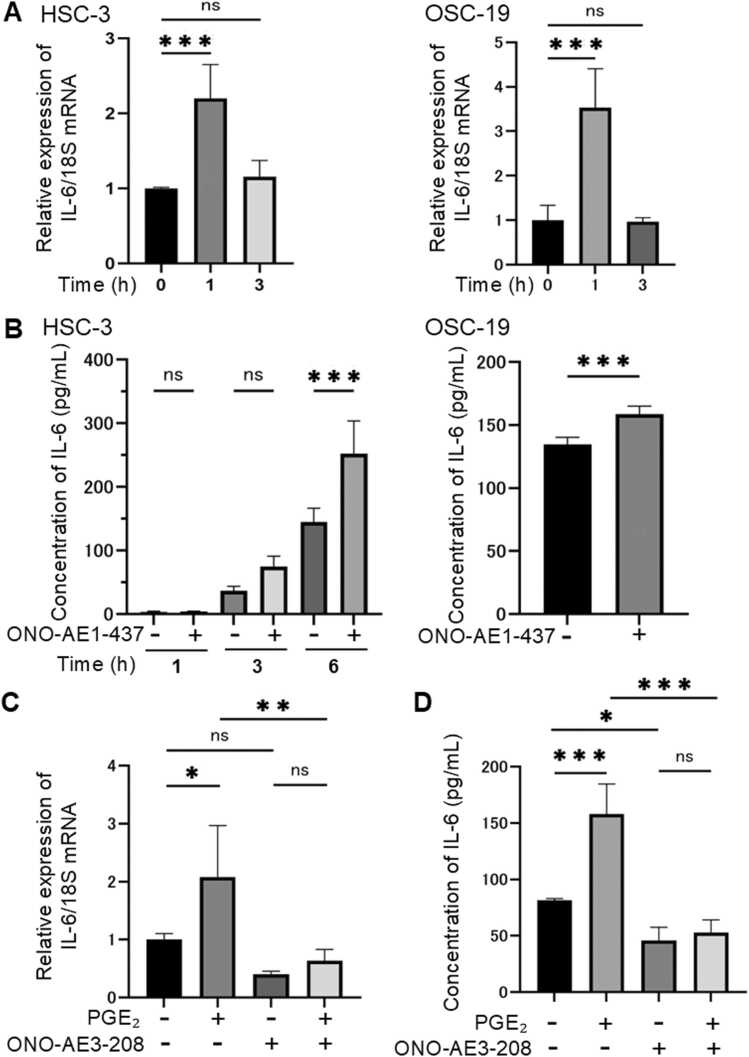
**EP4 stimulation increased IL-6 mRNA expression and protein secretion.** (A) IL-6 mRNA expression levels in HSC-3 and OSC-19 cells following stimulation with ONO-AE1–437 (1 μM). (B) Extracellular IL-6 protein secretion levels in HSC-3 cells following stimulation with ONO-AE1–437 (1 μM) for 1, 3, and 6 h, and in OSC-19 cells evaluated at 6 h. Time-course analyses were initially performed in HSC-3 cells, and the 6 h time point—at which EP4-induced changes were most prominent—was selected for subsequent evaluation in OSC-19 cells. (C) IL-6 mRNA expression in HSC-3 cells treated with PGE₂ (2 μM) or ONO-AE3–208 (1 μM) for 1 h. (D) IL-6 protein secretion in HSC-3 cells treated with PGE₂ (2 μM) or ONO-AE3–208 (1 μM) for 6 h. These results suggest that EP4 activation promotes both IL-6 mRNA expression and protein secretion. **A**–**D** Data are representative of *n* = 4 independent experiments.

To validate the RNA sequencing results, changes in IL-6 mRNA expression following ONO-AE1–437 stimulation were quantified using RT-qPCR. IL-6 mRNA expression was significantly increased in HSC-3 and OSC-19 cells after 1 h of ONO-AE1–437 stimulation (Fig. 2**A**). Additionally, changes in extracellular IL-6 protein secretion were evaluated using ELISA, which demonstrated that IL-6 secretion was increased in HSC-3 and OSC-19 cells following 6 h of EP4 stimulation (Fig. 2**B**). Based on preliminary time-course experiments performed prior to the main analyses, in which IL-6 secretion peaked at 6 h and declined at later time points, the 6 h time point was selected for subsequent ELISA analyses (Fig. S3). The presence of PGE₂ also increased IL-6 mRNA expression, and this increase was suppressed by the addition of ONO-AE3–208, a selective EP4 antagonist (Fig. 2**C**). Similarly, PGE₂ enhanced extracellular IL-6 secretion, which was also suppressed by ONO-AE3–208 (Fig. 2**D**). These results confirm that EP4 stimulation promotes IL-6 expression and secretion. In HSC-3 cells, IL-6 mRNA expression did not show a clear time-dependent increase after EP4 agonist stimulation, whereas IL-6 protein secretion increased in a time-dependent manner. Since IL-6 is known to act in an autocrine or paracrine manner to amplify its own production, it is likely that, in addition to de novo synthesis from mRNA, prolonged stimulation enhanced IL-6 secretion through this positive feedback mechanism. In addition to the two oral cancer cell lines described above, increased IL-6 expression after EP4 stimulation was confirmed in SCC-25 oral squamous cell carcinoma cells and HGnF normal human gingival fibroblasts (Fig. S4). The addition of exogenous PGE₂ increased both IL-6 mRNA expression and secretion, whereas treatment with the COX-2 inhibitor celecoxib reduced them. Furthermore, simultaneous treatment with celecoxib and PGE₂ partially restored IL-6 expression to approximately 1.5-fold of the control level (Fig. S5). These findings indicate that both endogenous and exogenous PGE₂ regulate IL-6 expression and secretion in HSC-3 cells. Importantly, celecoxib at the concentration used did not affect cell proliferation, confirming that the observed changes were not due to cytotoxic or growth-inhibitory effects.

### Upregulation of IL-6 expression by EP4 overexpression and further enhancement by EP4 stimulation

**Fig. 3:**
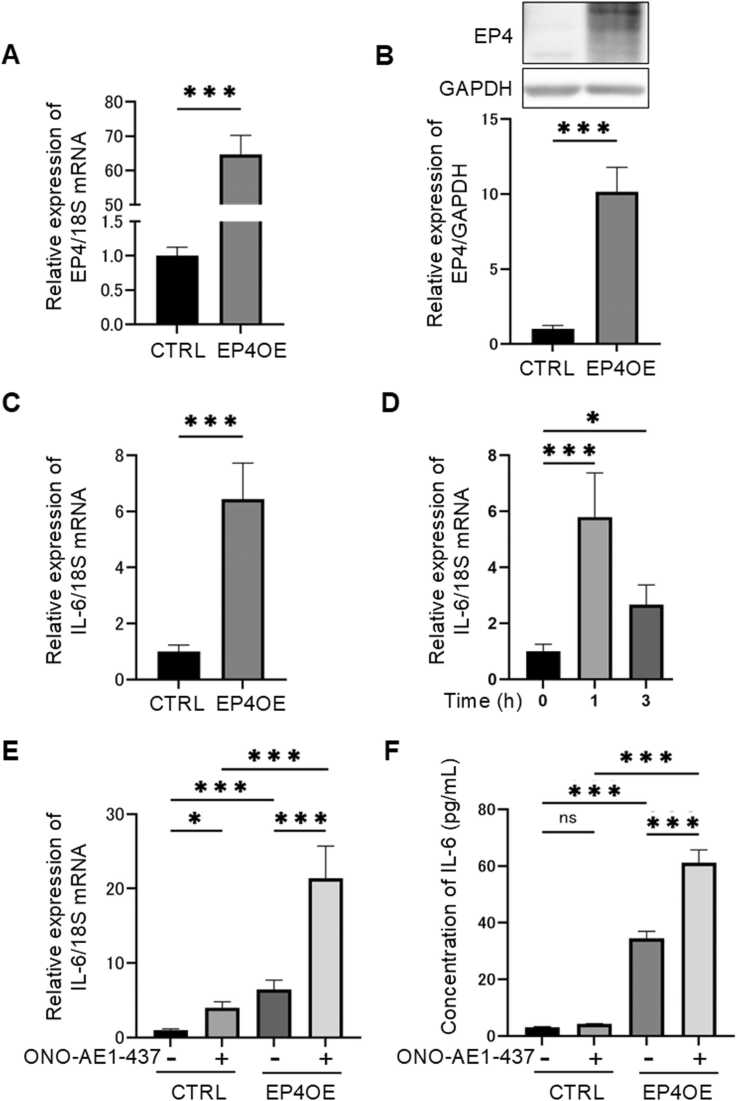
**EP4 overexpression increased IL-6 mRNA expression and protein secretion.** (A) EP4 mRNA expression levels in HSC-3 cells stably overexpressing EP4, established via lentiviral transduction. (B) EP4 protein expression in EP4-overexpressing cells. (C) IL-6 mRNA expression in EP4-overexpressing cells. (D) IL-6 mRNA expression in EP4-overexpressing cells following PGE₂ (2 μM) stimulation. (E) IL-6 mRNA expression in EP4-overexpressing cells following ONO-AE1–437 (1 μM) stimulation. (F) IL-6 protein secretion in EP4-overexpressing cells following ONO-AE1–437 (1 μM, 1 h) stimulation. **A**–**F** Data are representative of *n* = 4 independent experiments.

To further investigate the relationship between EP4 and IL-6, we established oral squamous cell carcinoma (HSC-3) cells with EP4 overexpression using lentiviral transduction. First, the overexpression of EP4 was confirmed by RT-qPCR and Western blotting, revealing that EP4 mRNA expression in EP4-overexpressing cells was approximately 60-fold higher than that in control cells (HSC-3 cells transduced with a control lentivirus without EP4 overexpression) (Fig. 3**A**). Additionally, EP4 protein levels were approximately 10-fold higher in EP4-overexpressing cells compared to control cells (Fig. 3**B**). Overexpression of EP4 did not affect cell proliferation within 24 h (Fig. S6). We then examined the basal IL-6 mRNA expression in EP4-overexpressing cells and found that it was approximately 6-fold higher than in control cells (Fig. 3**C**), indicating that EP4 overexpression leads to an increase in IL-6 expression as well as EP4 expression. Next, we evaluated the changes in IL-6 mRNA expression following stimulation with PGE₂ or ONO-AE1–437 in EP4-overexpressing cells. The addition of PGE₂ resulted in an approximately 6-fold increase in IL-6 mRNA expression compared to unstimulated cells (Fig. 3**D**). Stimulation with the ONO-AE1–437 for 1 h led to an approximately 3-fold increase in IL-6 mRNA expression (Fig. 3**E**). Furthermore, the extracellular IL-6 protein secretion was increased by approximately 2-fold (Fig. 3**F**). In EP4-overexpressing HSC-3 cells, both IL-6 mRNA expression and protein secretion were increased even under unstimulated conditions. We consider two possible explanations for this observation. First, EP4 overexpression may increase receptor density, leading to enhanced receptor–receptor interactions and elevated basal signaling activity. Second, the heightened sensitivity to endogenously produced PGE₂ may contribute to the enhanced IL-6 production. These findings suggest that overexpression of EP4 not only augments basal IL-6 expression but also further amplifies IL-6 induction upon EP4 stimulation.

These findings confirm that EP4 stimulation enhances IL-6 expression and secretion even in EP4-overexpressing cells, suggesting that higher levels of EP4 expression may further amplify EP4 stimulation-induced IL-6 expression.

### EP4 stimulation promotes cell adhesion via IL-6

**Fig. 4:**
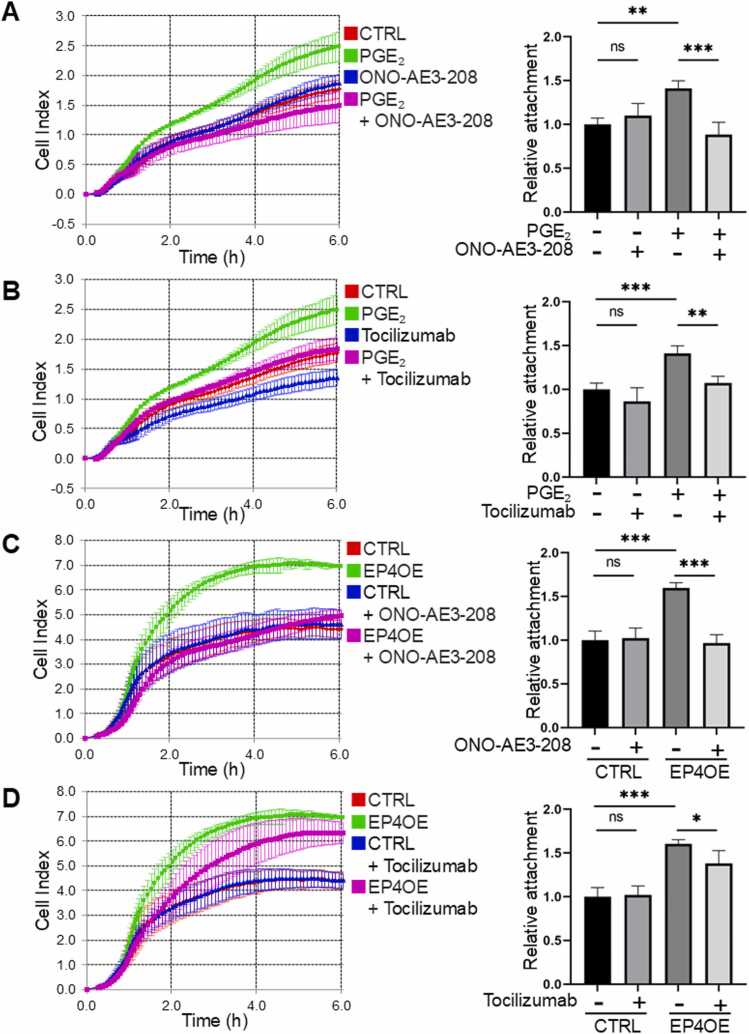
**EP4 promotes cell adhesion through IL-6 signaling.** Cell adhesion was assessed in real time using the xCELLigence RTCA system. The graph shows the adhesion capacity at 3 h after PGE₂ stimulation. (A) Effects of PGE₂ (2 μM) and ONO-AE3–208 (1 μM) on cell adhesion in HSC-3 cells. (B) Effects of PGE₂ (2 μM) and Tocilizumab (1 μg/mL) on cell adhesion in HSC-3 cells. (C) Effects of ONO-AE3–208 (1 μM) on cell adhesion in EP4-overexpressing cells. (D) Effects of Tocilizumab (1 μg/mL) on cell adhesion in EP4-overexpressing cells. **A**–**D** Data are representative of *n* = 4 independent experiments.

The time-dependent evaluation of cell adhesion was performed using HSC-3 cells. The results demonstrated that cell adhesion was enhanced in the presence of PGE₂, and this enhancement was suppressed by ONO-AE3–208 (Fig. 4**A**). Similarly, the addition of Tocilizumab suppressed the PGE₂-induced enhancement of cell adhesion (Fig. 4**B**). Tocilizumab did not affect cell proliferation (Fig. S7). In EP4-overexpressing cells, cell adhesion capacity was higher compared to the control group, and the addition of ONO-AE3–208 reduced this enhanced adhesion (Fig. 4**C**). Furthermore, the addition of Tocilizumab also suppressed the cell adhesion in EP4-overexpressing cells. These findings confirm that EP4 promotes cell adhesion through IL-6 signaling.

### EP4 promotes cell migration via IL-6

**Fig. 5:**
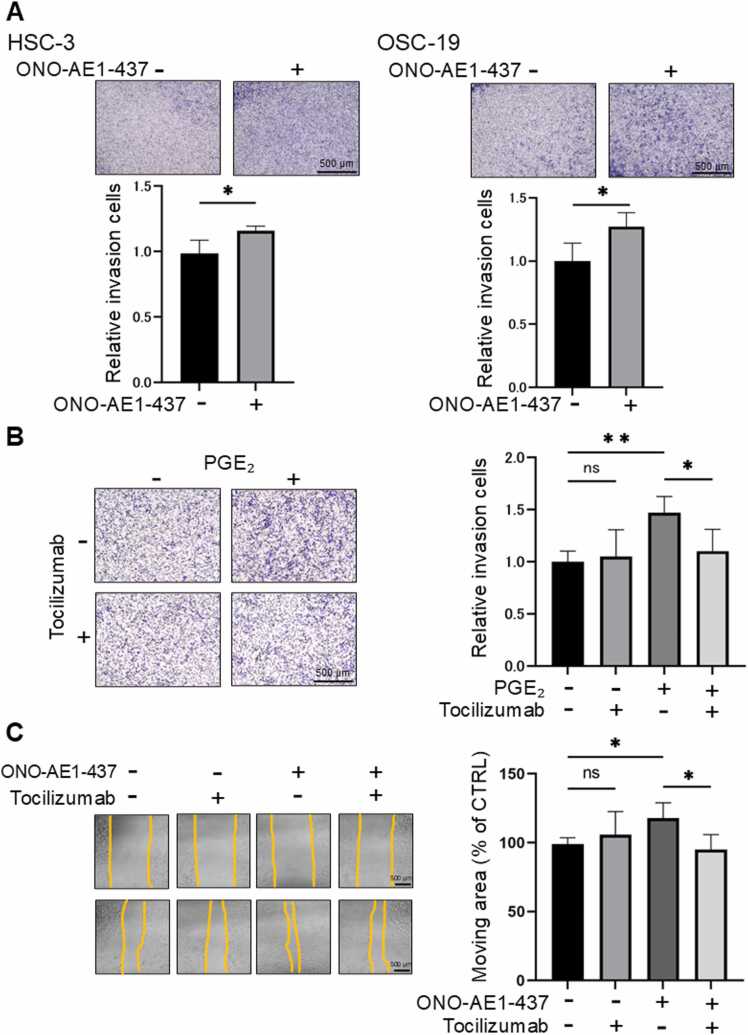
**EP4 regulates cell migration through IL-6 signaling.** (A) Effects of ONO-AE1–437 (1 µM) stimulation on cell migration in HSC-3 and OSC-19 cells. EP4 activation significantly enhanced cell migration in both cell lines. (B) Effects of PGE₂ (2 µM) stimulation with or without Tocilizumab (1 µg/mL) on cell migration in HSC-3 cells. PGE₂-induced migration was significantly suppressed by IL-6 inhibition. (C) Effects of ONO-AE1–437 (1 µM) stimulation with or without Tocilizumab (1 µg/mL) on cell migration in HSC-3 cells. EP4 stimulation induced migration was significantly suppressed by IL-6 inhibition. **A**–**C** Data are representative of *n* = 4 independent experiments.

Cell migration was evaluated using a transwell assay. Based on previous studies showing that treatment with EP4 agonists does not alter the proliferation of oral cancer cell lines even after 10 or 24 h, the scratch assay and transwell assay in this study were performed at 10 h and 24 h, respectively. Stimulation with ONO-AE1–437 enhanced the migration capacity of HSC-3 and OSC-19 cells (Fig. 5**A**). Additionally, the addition of PGE₂ increased cell migration, and this enhancement was suppressed by the addition of Tocilizumab (Fig. 5**B**). Furthermore, the suppression of EP4 stimulation-induced migration by IL-6 inhibition was also confirmed using a scratch assay (Fig. 5**C**). These results suggest that EP4 promotes the migration of cancer cells through IL-6 signaling.

## Discussion

In this study, we investigated the role of the EP4 signaling pathway in regulating IL-6 expression in OSCC cells and explored its potential involvement in modulating the ECM environment and related processes, such as cell migration and adhesion. Our findings demonstrated that activation of EP4 significantly increased both IL-6 mRNA expression and protein secretion in OSCC cells, whereas inhibition of EP4 attenuated these effects under PGE₂ stimulation. Moreover, EP4 overexpression further enhanced IL-6 production. Inhibition of IL-6 effectively suppressed PGE₂-induced increases in cell adhesion and migration, indicating that these cellular functions are mediated, at least in part, through the EP4-IL-6 axis (Fig. S8). Collectively, these results suggest that EP4 plays a pivotal role in modulating inflammatory cytokine production and related tumor cell functions in the tumor microenvironment of OSCC.

Recent literature shows that cancer cells can employ dynamic or transient adhesion mechanisms to facilitate migration rather than being impeded by adhesion itself. For example, collective migration retains cell–cell or cell–ECM adhesions in a regulated manner that contributes to invasive potential without loss of motility [36]. Moreover, cytoskeletal and adhesion molecule remodeling allow migration even when adhesion is elevated temporarily.

We further considered the downstream mechanisms by which PGE₂-EP4 signaling promotes IL-6 expression. Previous studies have demonstrated that EP4 activates multiple intracellular cascades, including the cyclic adenosine monophosphate/protein kinase A (cAMP/PKA), phosphoinositide 3-kinase/protein kinase B (PI3K/Akt), and mitogen-activated protein kinase (MAPK) pathways, all of which are involved in cytokine regulation [37], [38], [39], [40]. Consistent with this, our RNA-seq enrichment analysis showed significant upregulation of MAPK-related terms, suggesting that EP4 may enhance IL-6 transcription through MAPK-mediated activation of downstream transcription factors such as nuclear factor kappa B (NF-κB) and signal transducer and activator of transcription 3 (STAT3). Indeed, inhibition of the PGE₂-EP4 pathway has been reported to suppress MAPK signaling and reduce IL-6 expression in other inflammatory models [41]. Although we did not directly verify these pathways experimentally, our findings suggest a potential role of the EP4–MAPK–IL-6 axis contributes to the observed cytokine induction.

We acknowledge, however, that other EP receptors (EP1–3) may also contribute to IL-6 induction. In this study, we focused on EP4 because it is highly expressed in OSCC and because our previous work demonstrated its role in cell migration [10], [16]. Although agonists for EP1–3 are available, their selectivity is limited—for example, Sulprostone acts on both EP1 and EP3, making it difficult to dissect receptor-specific effects. Therefore, we restricted our analysis to EP4 in the present study. Nevertheless, the potential involvement of EP1–3 in IL-6 regulation remains an important question, which will be addressed in future investigations.

Previous studies have reported the involvement of EP4 in regulating inflammatory cytokines, particularly IL-6, in various cell types such as vascular smooth muscle cells and fibroblasts [22], [42]. EP4-mediated signaling has been shown to enhance IL-6 production via the TGF-β-activated kinase 1 (TAK1)–NF-κB/JNK/p38 pathway and to contribute to the progression of chronic inflammation and vascular lesions. The novelty of our study lies in the demonstration that the EP4-IL-6 pathway in cancer cells regulates cell adhesion and migration, thereby potentially promoting tumor progression. This extends the pathophysiological relevance of the EP4-IL-6 axis, previously described in inflammatory and vascular disease models, to tumor biology, highlighting its broader significance across disease contexts.

To our knowledge, this is the first study to demonstrate the entire PGE₂–EP4–IL-6–adhesion/migration axis in cancer cells. While previous reports have shown parts of this pathway—such as PGE₂-induced IL-6 expression in gastric cancer [43] and breast cancer [44], or PGE₂-mediated promotion of migration and adhesion in lung, liver, and prostate cancers [45], [46], [47], no prior study has experimentally linked EP4 activation to IL-6 induction and subsequent regulation of adhesion and migration. Thus, our findings establish a novel mechanism in OSCC that may also represent a broader paradigm relevant to other malignancies.

IL-6 is a well-established cytokine implicated in chronic inflammatory diseases and in the progression of many tumors [48], [49], [50]. IL-6 exerts diverse biological effects in cancer, including stimulation of tumor cell proliferation, inhibition of apoptosis, promotion of invasion and angiogenesis, modulation of immunity, and induction of metastasis and cancer cachexia. These pleiotropic functions enable IL-6 to act as a central mediator of the tumor microenvironment. In OSCC, our findings suggest that PGE₂–EP4–induced IL-6 may not only regulate cell adhesion and migration but also influence clinical outcomes by shaping a tumor-supportive microenvironment. Nevertheless, the specific contribution of the EP4–IL-6 axis to OSCC biology, particularly in cell adhesion and migration, remains to be elucidated.

Notably, EP4 agonist–induced IL-6 responses differed quantitatively between HSC-3 and OSC-19 cells. Although IL-6 mRNA induction was comparable or even greater in OSC-19 cells, the increase in extracellular IL-6 protein secretion was more pronounced in HSC-3 cells. Such discrepancies between mRNA expression and secreted protein levels are not uncommon and may reflect cell type–specific regulation at multiple post-transcriptional steps, including translation efficiency, protein stability, and secretory capacity. In addition, differences in basal endogenous IL-6 signaling may influence the dynamic range of inducible IL-6 secretion, resulting in a more robust apparent response in HSC-3 cells. Importantly, despite these quantitative differences, EP4 stimulation enhanced IL-6–dependent adhesion and migration in both cell lines, suggesting that functional outcomes depend not only on absolute cytokine levels but also on cellular sensitivity and signaling context.

In the tumor microenvironment, IL-6 functions as a pleiotropic cytokine that promotes inflammation, epithelial–mesenchymal transition (EMT), and extracellular matrix (ECM) remodeling, thereby enhancing tumor cell migration and invasion. These processes are particularly relevant in OSCC, where metastasis critically affects patient prognosis. Our findings that PGE₂–EP4 signaling enhances IL-6 expression and modulates ECM-related pathways suggest that this axis may contribute to poor prognosis in OSCC by facilitating tumor progression. Consistent with this, our RNA sequencing analysis also identified ECM-related gene modules associated with EP4 activity. Taken together, these results highlight the EP4–IL-6 axis as a potential therapeutic target for modulating the tumor microenvironment in OSCC.

In addition, IL-6 has been reported to upregulate adhesion molecules such as ICAM-1 and integrins and to activate Src–FAK signaling to induce EMT [51], [52], [53]. These mechanisms are consistent with our findings and suggest that the time-dependent increase in cellular adhesion observed after stimulation is mediated, at least in part, through IL-6–driven upregulation of adhesion molecules and activation of adhesion-related signaling pathways, rather than solely by strengthened interactions among pre-existing molecules.

Although our study primarily focused on elucidating the role of the PGE₂–EP4–IL-6 signaling axis using exogenous PGE₂ stimulation, it is possible that endogenous PGE₂ production also contributes to IL-6 induction and subsequent cellular responses. To examine the contribution of endogenous PGE₂ to IL-6 regulation in oral cancer cells, we analyzed the effects of exogenous PGE₂ and the COX-2 inhibitor celecoxib on IL-6 expression. PGE₂ treatment increased both IL-6 mRNA expression and protein secretion, whereas celecoxib treatment reduced IL-6 levels. Moreover, IL-6 expression was decreased by celecoxib treatment alone, suggesting that endogenous PGE₂ contributes to basal IL-6 production. Notably, IL-6 production was not completely abolished under COX-2 inhibition, and PGE₂-induced IL-6 expression was partially retained even when PGE₂ and celecoxib were applied simultaneously. These findings indicate that IL-6 production is regulated by both endogenous PGE₂–COX-2–dependent mechanisms and PGE₂-independent pathways. Thus, IL-6 expression in oral cancer cells appears to be maintained through layered regulatory mechanisms involving both endogenous and exogenous PGE₂ signaling. In addition, the expression level and signaling activity of IL-6 receptors may modulate OSCC cell responsiveness to IL-6, thereby influencing adhesion, migration, and the tumor-supportive microenvironment. The potential interplay between endogenous PGE₂ and IL-6 receptor signaling thus warrants further investigation in future studies.

Pharmacological inhibition of EP4, with its capacity to suppress IL-6 expression, presents a promising strategy not only for inflammatory diseases but also for tumors characterized by high IL-6 expression, such as OSCC. Tocilizumab, an anti-IL-6 receptor antibody, is already used in the treatment of various inflammatory and autoimmune conditions, as well as cytokine release syndrome (CRS) in the context of cancer immunotherapy [54]. While Tocilizumab has shown efficacy in preclinical studies for certain cancers, such as breast cancer, there are currently no reports of its clinical use or ongoing trials specifically for OSCC. Nevertheless, the potential for Tocilizumab or other IL-6-targeted therapies in OSCC remains, based on emerging evidence from other malignancies. It should be noted, however, that the limitations of monotherapy and the risk of infection, especially in immunocompromised cancer patients, must be considered. Future research should investigate the comparative and combinatory effects of ONO-AE3–208 and Tocilizumab in OSCC, as well as the detailed molecular mechanisms involved.

This study is mainly based on in vitro experiments, which cannot fully capture the complexity of in vivo responses. Nevertheless, the findings provide important mechanistic insights, and further validation using animal models and clinical specimens will strengthen the translational relevance of our results. Furthermore, cluster analysis using the MCODE algorithm in the RNA sequencing analysis identified three modules (IL-6, IL-11, and IL-13Rα) that were estimated to play central roles within the network. In this study, we focused only on IL-6, as it was most closely associated with both migration and adhesion. Investigation of the relationship and mechanisms involving EP4 and the other two modules will be addressed in future studies. In our analysis, CSF1 (colony-stimulating factor 1) was annotated to migration/adhesion-related terms, suggesting a potential role—together with IL-6—in regulating adhesion and migratory functions within the tumor microenvironment. CSF1 is a cytokine that signals through CSF1R and regulates monocyte/macrophage biology and remodeling of the tumor microenvironment. However, CSF1 was not included in the MCODE-defined module in this dataset and was therefore not analyzed further within the module-based framework. The contribution of CSF1 outside the IL-6–centered module warrants future investigation.

In conclusion, this study demonstrates that the EP4–IL-6 pathway constitutes a novel mechanism regulating cell migration and adhesion in OSCC. These findings provide new insights into the molecular basis of tumor progression.

## CRediT authorship contribution statement

**Akane Nagasako:** Methodology. **Yu Iida:** Validation, Software, Methodology, Formal analysis, Data curation, Conceptualization. **UMEMURA MASANARI:** Writing – review & editing, Writing – original draft, Visualization, Supervision, Project administration, Methodology, Investigation, Funding acquisition, Formal analysis, Data curation, Conceptualization. **Yoshihiro Ishikawa:** Writing – review & editing, Conceptualization. **Wakana Fukae:** Writing – original draft, Visualization, Validation, Software, Resources, Methodology, Data curation, Conceptualization. **Kenji Mitsudo:** Writing – review & editing, Supervision, Investigation. **Utako Yokoyama:** Writing – review & editing, Investigation. **Takayuki Fujita:** Writing – review & editing, Investigation. **Oguri Senri:** Writing – review & editing, Conceptualization. **Kagemichi Nagao:** Supervision, Investigation. **Chihiro Hayashi:** Methodology, Conceptualization. **Michiko Endo:** Methodology. **Soichiro Ishikawa:** Investigation, Data curation, Conceptualization.

## Ethics statement

This study was conducted in accordance with the regulations for recombinant DNA experiments, and the experimental protocol was approved by Yokohama City University School of Medicine (approval number: F-D-24–17–2).

## Consent for publication

All authors read and approved the final manuscript for publication.

## Publisher's note

Springer Nature remains neutral with regard to jurisdictional claims in published maps and institutional affiliations.

## Declaration of generative AI and AI-assisted technologies in the writing process

During the preparation of this study, the author used ChatGPT 4o for English writing and proofreading. After using this tool, the author carefully reviewed and edited the content as needed, and took full responsibility for the content of the published article.

## Funding information

This work was supported in part by the 10.13039/501100001691Japan Society for the Promotion of Science (22K06928, 22K10154, 23K25180, 23K07483) (to M.U.).

## Declaration of Competing Interest

The authors declare the following financial interests/personal relationships which may be considered as potential competing interests:Masanari Umemura reports financial support was provided by The Japan Society for the Promotion of Science. If there are other authors, they declare that they have no known competing financial interests or personal relationships that could have appeared to influence the work reported in this paper.

## Footnotes

Appendix A Supplementary data associated with this article can be found in the online version at doi:10.1016/j.jphyss.2026.100057.
