# Supplementary material for: EP4 stimulation promotes cell adhesion and migration via IL-6 signaling in oral squamous cell carcinoma
Source: J Physiol Sci. 2026 Jan 15;76(1):100057. doi: 10.1016/j.jphyss.2026.100057 (PMC12859506; doi:10.1016/j.jphyss.2026.100057)
Supplement: Supplementary file 2 — Supplementary material [file mmc2.docx]

**Supplemental Figures**

**EP4 Stimulation Promotes Cell Adhesion and Migration via IL-6 Signaling in Oral Squamous Cell Carcinoma**

**Short title**

EP4 Enhances Adhesion and Migration via IL-6


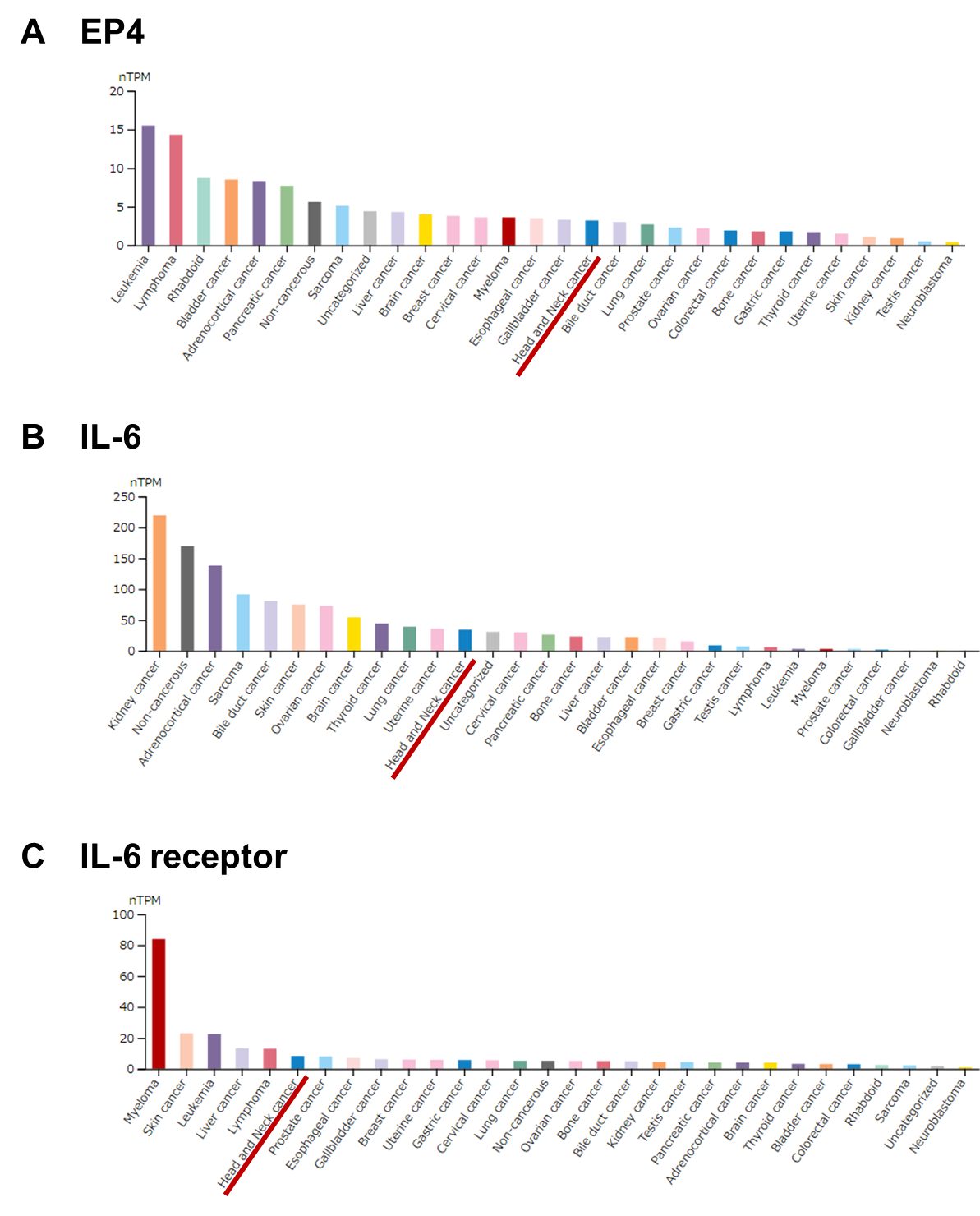


**Supplementary Figure 1. EP4 and IL-6 RNA expressions in head and neck cancer cell lines according to The Human Protein Atlas.**

(A) EP4 expression in head and neck cancer cell lines is the 17th highest among 30 cancer types.

(B) IL-6 expression in head and neck cancer cell lines is the 12th highest among 30 cancer types.

(C) IL-6 receptor expression in head and neck cancer cell lines is the 6th highest among 30 cancer types.


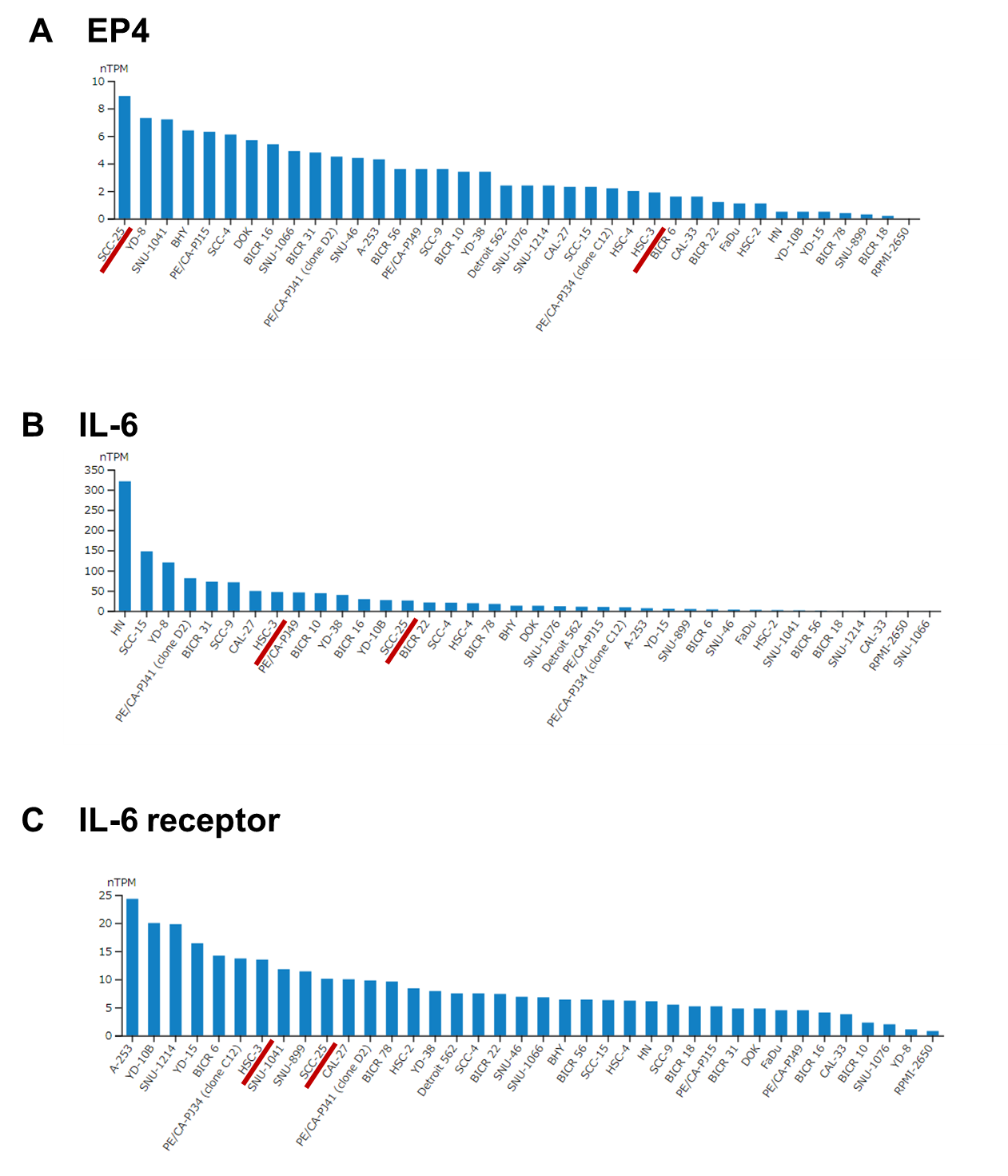


**Supplementary Figure 2. EP4 and IL-6 RNA expression in HSC-3 and SCC-25 cell lines according to The Human Protein Atlas.**

(A) EP4 expression is the 26th highest in HSC-3 and the highest in SCC-25 among 38 head and neck cancer cell lines.

(B) IL-6 expression is the 8th highest in HSC-3 and the 14th highest in SCC-25 among the same 38 cell lines.

(C) IL-6 receptor expression is the 7th highest in HSC-3 and the 10th highest in SCC-25 among the same 38 cell lines.

**
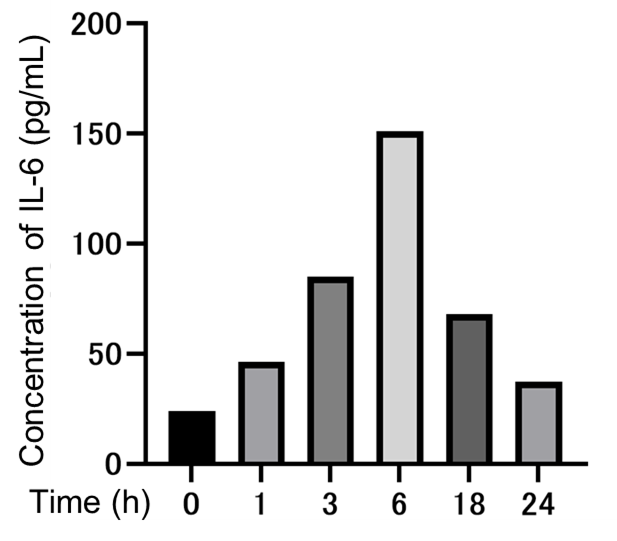
**

**Supplementary Figure 3. Preliminary time-course analysis of IL-6 secretion in HSC-3 cells following EP4 stimulation.**

HSC-3 cells were stimulated with the EP4 agonist ONO-AE1-437 (1 μM), and extracellular IL-6 levels were measured at the indicated time points. IL-6 secretion peaked at 6 h after stimulation. These data represent a preliminary experiment (n = 1).

　　　　　
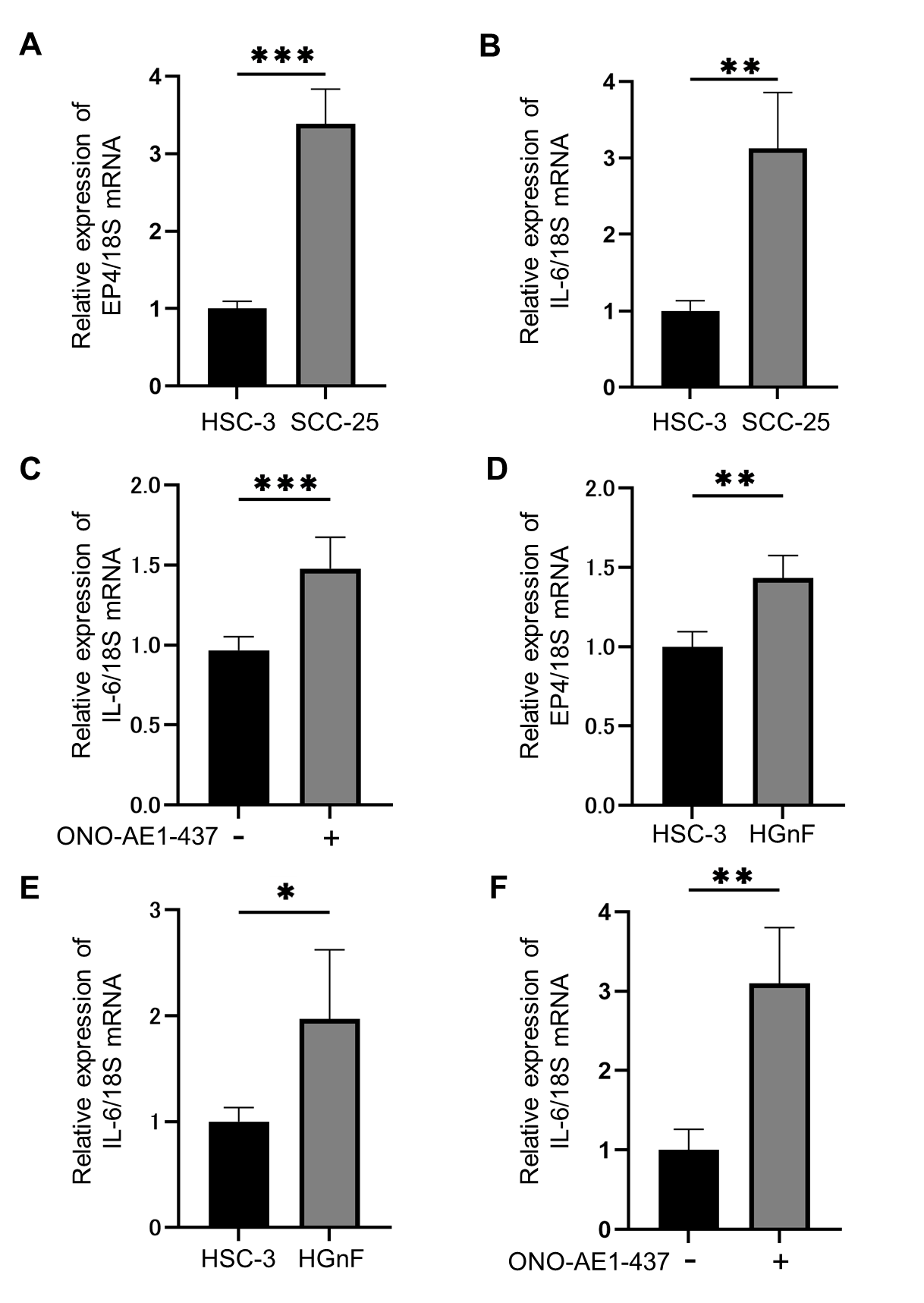


**Supplementary Figure 4. EP4 stimulation increases IL-6 mRNA expression in oral cancer cells (SCC-25) and normal cells (HGnF).**

(A) Comparison of EP4 mRNA expression levels between HSC-3 and SCC-25 cells.

(B) Comparison of IL-6 mRNA expression levels between HSC-3 and SCC-25 cells.

(C) IL-6 mRNA expression in SCC-25 cells following ONO-AE1-437 (EP4 agonist) stimulation (1 µM, 1 h).

(D) Comparison of EP4 mRNA expression levels between HSC-3 and HGnF cells.

(E) Comparison of IL-6 mRNA expression levels between HSC-3 and HGnF cells.

(F) IL-6 mRNA expression in HGnF cells following ONO-AE1-437 (EP4 agonist) stimulation (1 µM, 1 h).

**
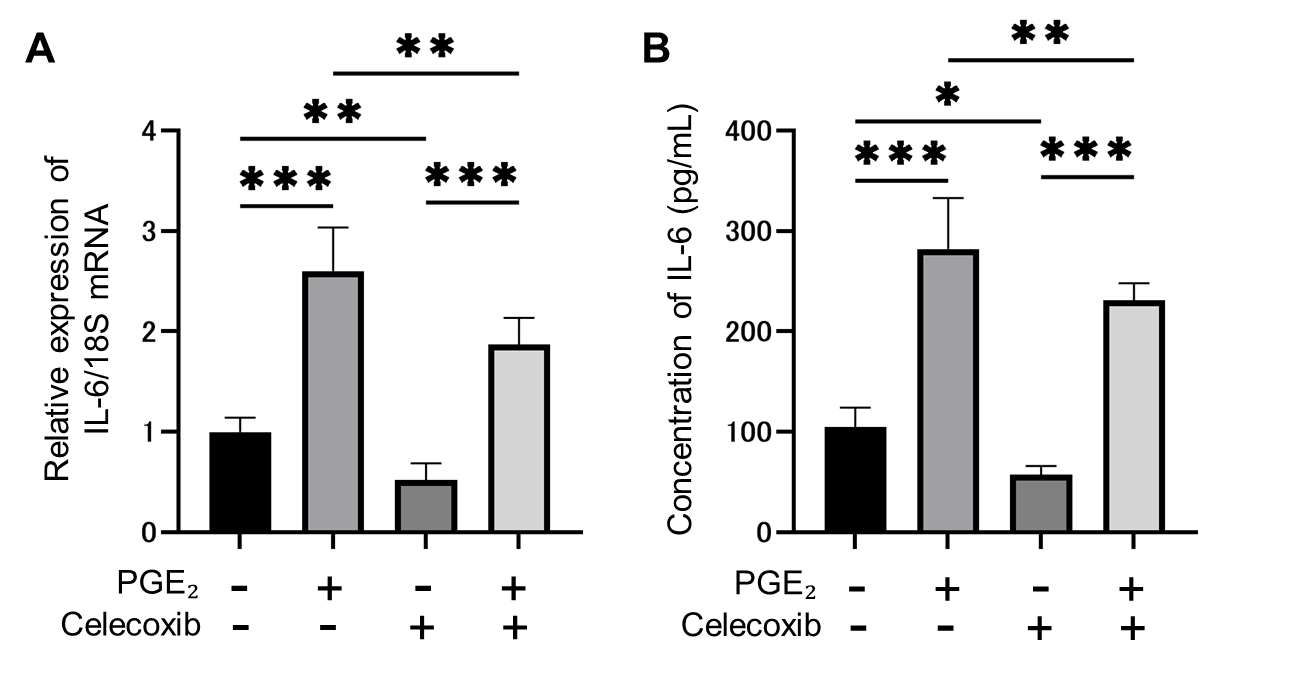
**

**Supplementary Figure 5.** **Endogenous PGE₂ and exogenous PGE₂ promote IL-6 mRNA expression and secretion.**

(A) Endogenous PGE₂ and exogenous PGE₂ promote IL-6 mRNA expression.

(B) Endogenous PGE₂ and exogenous PGE₂ promote IL-6 secretion.

　　　　　
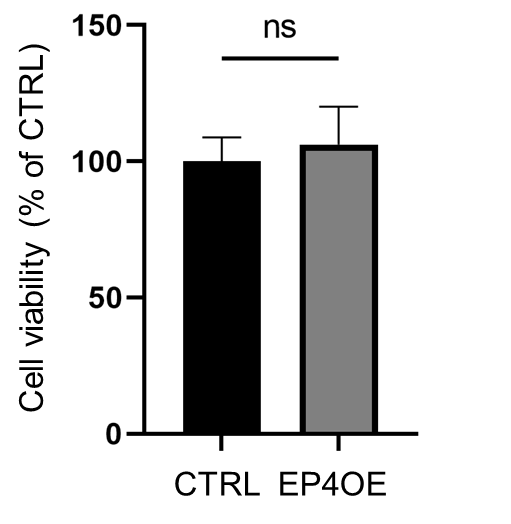


**Supplementary Figure 6. Overexpression of EP4 did not affect cell proliferation.**

Overexpression of EP4 did not affect cell proliferation as a result of CCK-8 (n=14).


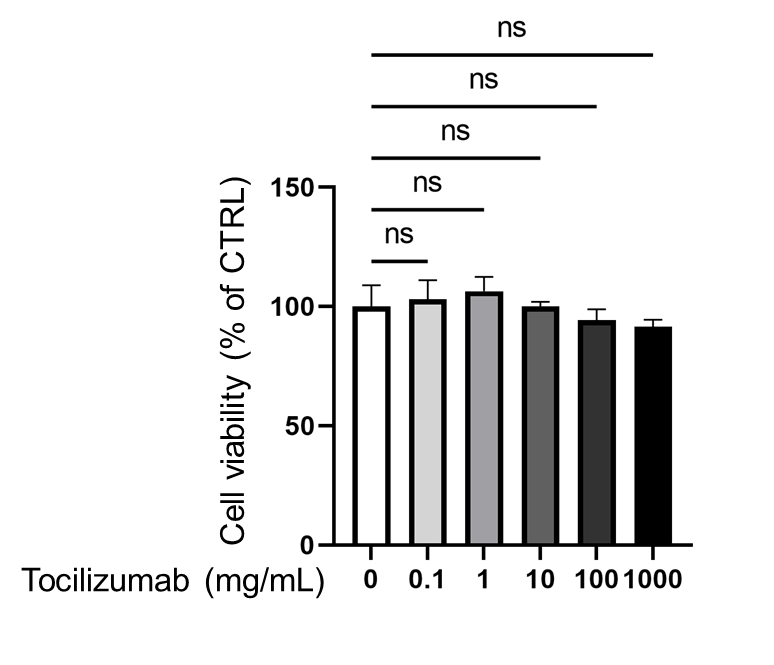


**Supplementary Figure 7. Tocilizumab did not affect cell proliferation.**

Tocilizumab (IL-6 inhibitor) did not affect cell proliferation as a result of CCK-8 (n=6).


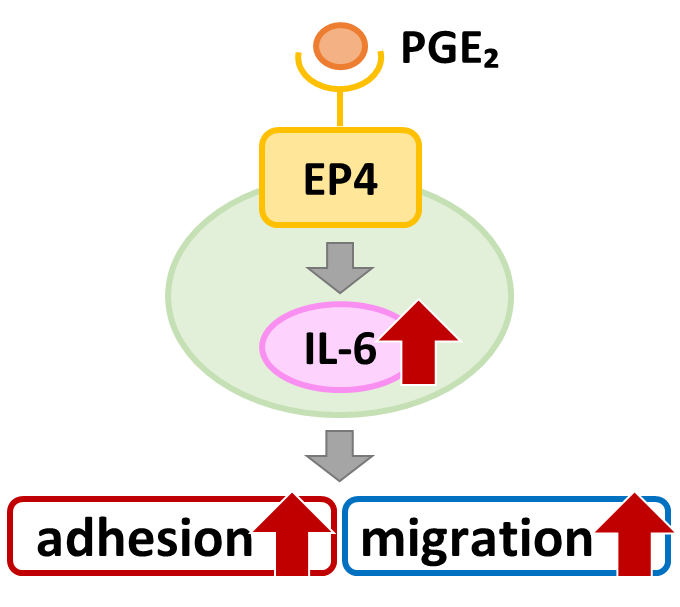


**Supplementary Figure 8. PGE₂ promotes cell adhesion and migration via EP4 and IL-6.**

Proposed mechanism by which PGE₂ enhances cell adhesion and migration through EP4 and IL-6 signaling in HSC-3 cells.


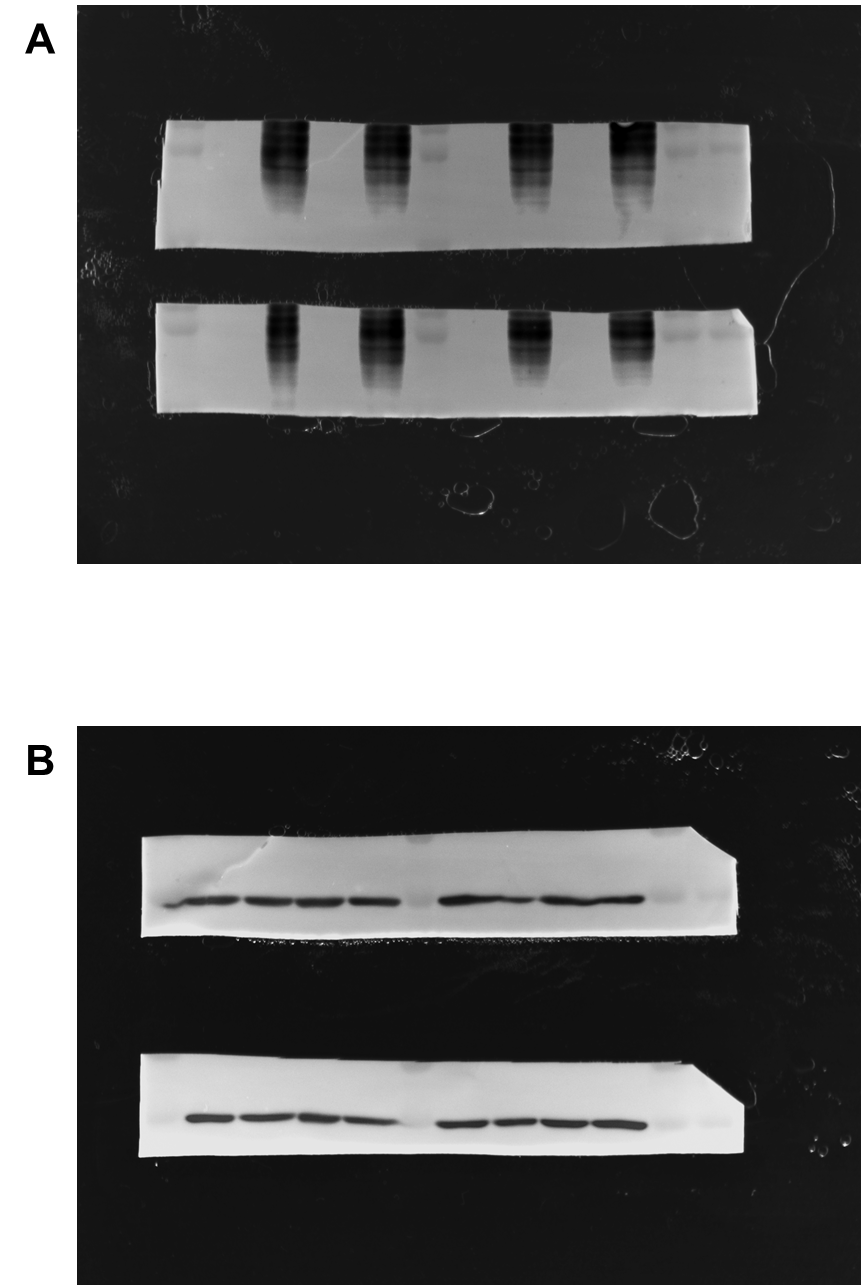


**Supplementary Figure 9. Full images of membranes corresponding to Fig. 3B.**

(A) Original membrane image for EP4.

(B) Original membrane image for GAPDH.
